# Tapping the Potential of Multimodal Non-invasive Brain Stimulation to Elucidate the Pathophysiology of Movement Disorders

**DOI:** 10.3389/fnhum.2021.661396

**Published:** 2021-05-12

**Authors:** Sakshi Shukla, Nivethida Thirugnanasambandam

**Affiliations:** National Brain Research Centre (NBRC), Manesar, India

**Keywords:** non-invasive brain stimulation, movement disorder, transcranial magnetic stimulation, magnetic resonance imaging, positron emission tomograghy, essential tremor, Parkinson’s disease, dystonia

## Abstract

This mini-review provides a detailed outline of studies that have used multimodal approaches in non-invasive brain stimulation to investigate the pathophysiology of the three common movement disorders, namely, essential tremor, Parkinson’s disease, and dystonia. Using specific search terms and filters in the PubMed^®^ database, we finally shortlisted 27 studies in total that were relevant to this review. While two-thirds (Brittain et al., 2013) of these studies were performed on Parkinson’s disease patients, we could find only three studies that were conducted in patients with essential tremor. We clearly show that although multimodal non-invasive brain stimulation holds immense potential in unraveling the physiological mechanisms that are disrupted in movement disorders, the technical challenges and pitfalls of combining these methods may hinder their widespread application by movement disorder specialists. A multidisciplinary team with clinical and technical expertise may be crucial in reaping the fullest benefits from such novel multimodal approaches.

## Introduction

**FIGURE 1:**
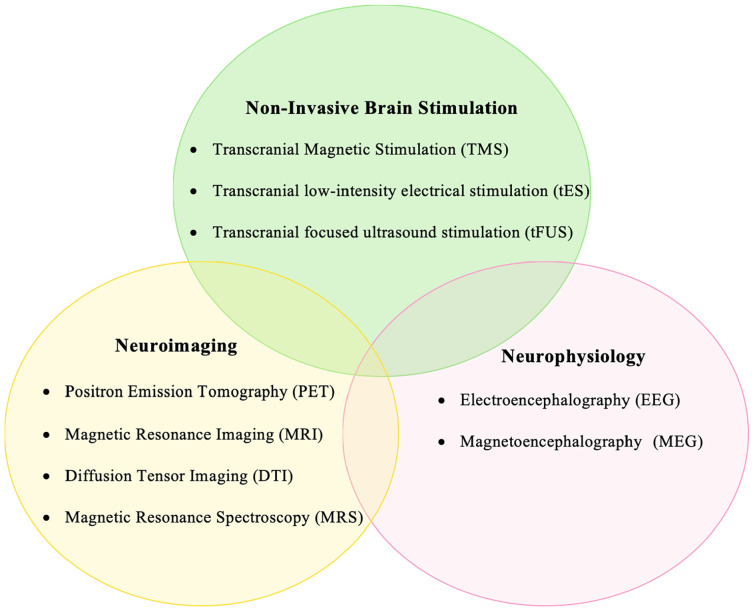
Diagram showing the different non-invasive brain stimulation, neuroimaging, and neurophysiological techniques that can be integrated to investigate the pathophysiology of movement disorders.

Movement disorders are a class of neurological syndromes that are characterized by uncontrollable, abnormally increased, or decreased movements. They rank among the most common neurological diseases with a prevalence of about 28% in middle-aged and elderly populations (Wenning et al., 2005). The most common movement disorders include essential tremor, Parkinson’s disease, and dystonia (Wichmann, 2018). These disorders are often progressive, increasing in severity, thereby causing considerable disability over time. Little is known about the pathophysiology of these disorders, and a lot still remains to be explored. Understanding their pathophysiological mechanisms is crucial to developing novel diagnostic tools and therapeutic strategies. Non-invasive brain stimulation (NIBS) methods have played a key role in understanding the neurophysiological mechanisms underlying clinical phenomena in patients with movement disorders (Quartarone, 2013; Rothwell, 2007; Ugawa et al., 2020). Transcranial magnetic stimulation (TMS), is a painless, non-invasive brain stimulation technique that has been used to study motor physiology for over three decades (Hallett, 2000; Chail et al., 2018). Novel TMS paradigms have been developed over the years to unravel the physiology of human motor control in health and disease. TMS has contributed significantly to our understanding of the altered neurophysiology in patients with movement disorders, for example, in dissociating the neural networks causing essential and parkinsonian tremors (Hanajima et al., 2016; Shih and Pascual-Leone, 2017), in identifying the impaired cortical inhibition in dystonia and Parkinson’s disease (Rothwell, 2007), and in differentiating the diagnosis of organic and functional dystonia (Quartarone et al., 2009). Recently, other NIBS methods such as transcranial direct/alternating current stimulation (tDCS/tACS) have also gained attention (Antal et al., 2017). These techniques, although in their infancy, offer great promise in exploring the pathophysiology of movement disorders. A more efficient approach is to combine the use of different NIBS with neuroimaging/neurophysiological methods such as Positron emission tomography (PET), magnetic resonance imaging (MRI), and magneto-/electro-encephalography (M/EEG). The knowledge gained from such a multimodal approach could be manifold as compared with employing individual techniques. Figure 1 shows the different non-invasive brain stimulation and neuroimaging/neurophysiological methods that can be combined effectively for studying movement disorders.

In this mini review, we describe the different multimodal NIBS approaches that have been used to study the pathophysiology of movement disorders and also discuss the immense potential such an approach offers in enhancing our understanding of these disorders. We discuss the three most common movement disorders—essential tremor, Parkinson’s disease, and dystonia—and how multimodal NIBS studies have enhanced our knowledge and understanding of these disorders. We also propose future research directions for movement disorder specialists by discussing the scope of some of the most recent advancements in the field of NIBS.

## Materials and Methods

This mini-review includes research studies conducted to date that have used a multimodal approach using non-invasive brain stimulation techniques for movement disorders such as Parkinson’s disease, dystonia, and essential tremor. Related research studies were searched on the PubMed^®^ database^1^ using the advanced search builder feature. Within the “All fields” category, search terms were added in the following pattern: ([(Disease name) OR (Disease Acronym)] AND [(Non-invasive brain technique) OR (Acronym)] AND [(other technique) OR (Acronym)]). An example of a search terms sequence used for Parkinson’s Disease was: “([(Parkinson’s Disease) OR (PD)] AND [(Transcranial Magnetic Stimulation) OR (TMS)] AND [(Magnetic Resonance Imaging) OR (MRI)]).” Similarly, various different combinations of NIBS techniques and other modalities were searched for each of the three diseases. A total of 1,416, 506, and 86 results were obtained for PD, Dystonia, and ET, respectively. Out of the total number of results, studies with a multimodal approach were filtered out for each disease, which counted about 86 for PD, 14 for dystonia, and 3 for ET. Furthermore, case reports and review articles were excluded. Finally, the number of research articles included in qualitative synthesis were 18, 6, and 3 for PD, dystonia, and ET, respectively. The relevant papers were then thoroughly read and reread, with the aim of determining key methods used and their advantages. Their results were analyzed as to how different multimodal approaches help in elucidating the pathophysiology of movement disorders.

### Essential Tremor

Essential tremor (ET) is a brain disorder that causes uncontrollable and rhythmic shaking of one or more body parts, most commonly the upper limbs. It is the most common movement disorder affecting approximately 4% of adults over the age of 40 years (Zesiewicz et al., 2010). Although about half of the patients with ET have a positive family history, an equal percentage of them are idiopathic (Elble, 2013). The diagnosis is often confused with Parkinsonian tremor or dystonic tremor (Tarakad and Jankovic, 2018; Panyakaew et al., 2020). It is important to differentiate among these tremor types for their effective management. However, little is known about the pathophysiology underlying tremors of different etiologies. We found only a few studies in literature that employed multimodal NIBS to investigate ET. A notable study by Popa et al. (2013) showed that low frequency (1 Hz) repetitive TMS (rTMS) applied over bilateral posterior cerebellar cortices for a week successfully reduced the overall amplitude of the tremors. Furthermore, this clinical effect, which lasted for about 3 weeks, was associated with an improvement in the functional connectivity of the cerebello-thalamo-cortical network and there was no change in the functional connectivity within other networks such as the default mode network. These results clearly indicate that ET may be caused by abnormal connectivity in the cerebello-thalamo-cortical circuit and that suppressing the excitability of the bilateral cerebellum using rTMS could be an effective treatment option for patients with severe ET. Another study by Lu et al. (2016) investigated the effect of an associative plasticity-inducing TMS protocol, on the structural connectivity of the corticospinal tract in patients with ET and Parkinson’s disease with intention tremor. The authors found that the microstructure of the corticospinal tract was intact in both these patient groups, suggesting that the corticospinal tract may not be relevant to the deficient motor plasticity seen in them (Lu et al., 2016). Fox and colleagues showed, using resting-state functional connectivity MRI, that NIBS was effective only if applied to cortical regions with good functional connectivity to effective deep brain stimulation sites. This reiterates the importance of combining information from neuroimaging to effectively target NIBS.

ET, although the most common movement disorder, has not been studied enough using multimodal approaches. Novel NIBS methods, especially transcranial alternating current stimulation (tACS) administered in a closed-loop pattern, have proven to be beneficial in reducing the tremor amplitude in ET patients (Brittain et al., 2013; Schreglmann et al., 2021). Investigating the pathophysiology of ET using concurrent tACS with M/EEG is likely to shed light on the contribution of key neurophysiological mechanisms in the disease process.

### Parkinson’s Disease

Parkinson’s disease (PD) is a neurodegenerative disorder characterized by loss of dopaminergic neurons in the substantia nigra of the basal ganglia, manifesting itself as bradykinesia, rigidity, rest tremor, postural imbalance, and other non-motor features (Poewe et al., 2017). Although some of the primary disease symptoms respond to dopamine supplementation, not all features of the disease are mediated by a dopaminergic deficit (Miguelez et al., 1460). The neurophysiological mechanisms underlying several of the clinical features of PD still remain to be explored (Jankovic and Sherer, 2014). Here we discuss some of the multimodal NIBS approaches that have been employed to study the pathophysiology of PD.

Positron emission tomography (PET) is a functional neuroimaging technique that uses radioactive tracer elements to visualize and measure changes in cerebral blood flow resulting from metabolic processes. The combination of PET and TMS to study PD was already used as recently as 2001. Strafella et al. (2001) used [^11^C] raclopride (a dopamine receptor ligand) to measure the dopamine release in the human striatum following rTMS to the dorsolateral prefrontal cortex (DLPFC). rTMS was also applied to the occipital region as a control. Low frequency (1 Hz) rTMS to DLPFC but not to the occipital cortex decreased the [^11^C] raclopride binding potential in the ipsilateral caudate nucleus, implying that activation of corticostriatal fibers originating in the DLPFC is involved in dopamine release at the respective projection site in the striatum. In a follow-up study, it was also shown that rTMS in the primary motor cortex induced dopamine release in the ipsilateral putamen (Strafella et al., 2003). Furthermore, the same combination of TMS and PET was used to identify potential differences in corticostriatal dopamine release between the symptomatic and presymptomatic hemispheres. A frequency of 10 Hz rTMS to the primary motor cortex on the symptomatic hemisphere revealed less striatal dopamine release; however, with a significantly larger cluster size. This spatially enlarged area of dopamine release in the symptomatic hemisphere possibly indicates a loss of functional segregation and abnormal corticostriatal transmission in early PD (Strafella et al., 2005). Sacheli et al. (2019) conducted a multimodal study combining PET and fMRI during TMS to investigate the effects of exercise in PD patients. They aimed to evaluate the effect of exercise on dorsal striatal dopamine release and the ventral striatal response to reward anticipation. The results of this study showed that exercise in PD patients enhances the dopaminergic function and reward-related responsivity in both nigrostriatal and mesolimbic projections, thereby contributing to improvements in motor function, mood, and apathy. Fregni et al. (2006) also studied depression in PD using rTMS and SPECT, which measured the changes in regional cerebral blood flow (rCBF). They reported significantly lower rCBF in the left prefrontal, posterior cingulate gyrus, and left insula and right parietal cortex in PD patients as compared with healthy controls. Furthermore, rTMS improved depression significantly associated with increased rCBF in the posterior cingulate cortex, indicating that depression in PD is associated with a dysfunction of the fronto-limbic network connectivity that can be effectively modulated by rTMS.

Magnetic resonance spectroscopy (MRS) is used to measure the different metabolite concentrations in the brain. Flamez et al. (2019) applied 1Hz rTMS over the right pre-supplementary motor area (SMA) and assessed the change in choline/creatine ratio in PD patients. They found that low frequency (1 Hz) rTMS significantly increased the choline/creatine ratio but only when disease duration was taken into account, that is, the shorter the duration of disease, the stronger the observed effects were. This implies that in the early stages of PD, membrane turnover at the pre-SMA could still be influenced by a single session of rTMS suggesting that at least some brain plasticity is preserved. In addition to primary motor deficits, PD patients have abnormal sensory processing (Hwang et al., 2016). Functional MRI combined with somatosensory stimulation in the form of vibrotactile stimulation (Nelson et al., 2018) or laser-induced nociceptive stimulation (Petschow et al., 2016) revealed deficient activation of the somatosensory cortex in the former and the nodes of the central pain matrix in the latter for PD patients. A TMS-fMRI study revealed that depression in PD patients may result from increased activity of the medial prefrontal cortex (Cardoso et al., 2008).

One of the recent advances in the field of NIBS is the successful coupling of TMS and EEG (Tremblay et al., 2019). Both these techniques have an excellent temporal resolution that makes it an excellent combination to examine the neurophysiological processes that take place within a millisecond. Although the field of TMS-EEG is still in its infancy, it has already been used successfully to study PD. Concurrent TMS-EEG has yielded a wealth of new information on the pathophysiology of PD, which neither of the techniques did when used individually. One of the earliest TMS-EEG studies in PD patients by Casarotto et al. (2019) showed that levodopa intake increased the cortical reactivity over the SMA ipsilateral to the more affected putamen. TMS-EEG has also been used to study the role of the motor cortex in re-emergent tremor in PD (Leodori et al., 2020). TMS over the primary motor cortex caused stable resetting of the re-emergent tremor, which suggests that the primary motor cortex is a crucial node in the cortico-subcortical network that generates a re-emergent tremor and is more than just an output region. TMS-induced oscillatory activity was studied by Van Der Werf et al. (2006) in PD patients who underwent unilateral thalamotomy. They reported that the TMS-induced beta oscillatory power was lower in the operated hemisphere, indicating that thalamotomy successfully reduced the pathological beta oscillations in the cortico-subcortical network in PD patients. NIBS methods other than TMS have also been used in combination with neuroimaging to study PD. Pereira et al. (2013) integrated tDCS and fMRI to study phonemic and semantic fluency, another non-motor feature in PD patients. They observed that tDCS over DLPFC, but not the temporo-parietal cortex, enhanced functional connectivity in the verbal fluency and the deactivation task-related network.

Levodopa-induced dyskinesias (LID) are a common complication of PD (Espay et al., 2018). Although there are a few hypotheses, the exact etiology of LID is unknown (Pandey and Srivanitchapoom, 2017). A resting-state fMRI revealed impaired functional connectivity between the right inferior frontal cortex, contralateral primary motor cortex, and ipsilateral putamen with levodopa intake. Furthermore, continuous theta-burst stimulation applied over the right inferior frontal cortex reduced dyskinesia severity suggesting that the pathophysiological mechanisms underlying LID may extend beyond the basal ganglia and possibly involve neural networks centered on the inferior frontal cortex (Cerasa et al., 2015). Another study by Brusa et al. (2012) showed improvement of dyskinetic symptoms associated with a reduction of ^18^fluorodeoxyglucose (FDG) metabolism in the cerebellum following 1 week of daily bilateral cerebellar continuous theta-burst stimulation. These findings suggest that the interventional TMS protocol modulated the activity of neural pathways connecting the cerebellar cortex with deep cerebellar nuclei.

In summary, PD has been the most investigated movement disorder using multimodal NIBS approaches. These studies have been able to successfully identify novel neurophysiological mechanisms that are likely to contribute to the pathophysiology of PD.

### Dystonia

Dystonia is a complex and highly variable movement disorder characterized by involuntary muscle contractions that cause slow, repetitive, twisting movements or abnormal postures affecting any body part such as the arms, legs, trunk, face, or vocal cords (Phukan et al., 2011). The etiology of primary/idiopathic dystonia is not known; hence, treatment options are also very limited (Balint et al., 2018). The most common form of idiopathic dystonia is focal (involving a single body part) and therefore is also the most studied form of dystonia, using multimodal NIBS. Neuroimaging studies suggest that dystonia is likely to be a disorder of abnormal functional connectivity (van Wijk et al., 2017). Bharath et al. (2015) using fMRI showed that resting-state functional connectivity within the network comprising the contralateral premotor cortex, intraparietal sulcus, cerebellum, bilateral thalamus, putamen, globus pallidus, and the bilateral supplementary motor area was lower in patients with writer’s cramp (WC) and that low-frequency (1 Hz) rTMS administered over the primary motor cortex-improved functional connectivity within the motor network. The authors, using a combination of rTMS and fMRI, concluded that WC is probably a network disorder involving subcortical and trans-hemispheric brain regions with widespread dysfunction much larger than is clinically evident. From several studies, we know that impaired sensorimotor integration also plays a role in the pathophysiology of dystonia. Schneider et al. (2010) applied high-frequency rTMS over the left primary somatosensory cortex in WC patients and measured its effects on tactile discrimination accuracy and hemodynamic activity. Their findings revealed that tactile discrimination in patients was lower than that in healthy controls and that 5 Hz rTMS did not improve the condition. On fMRI, rTMS-induced improvement in discrimination in healthy controls that was associated with enhanced basal ganglia activation was absent in WC patients. This may reflect impaired basal ganglia-somatosensory connectivity in WC patients. An rTMS interventional study (Havrankova et al., 2010) also supports the hypothesis that clinical improvement in writer’s cramp patients following multiple rTMS sessions over the primary somatosensory cortex is associated with enhanced connectivity in the sensorimotor network comprising the primary somatosensory, supplementary motor, and posterior parietal cortices. Apart from the primary motor and somatosensory cortices, the multimodal approach enabled us to study the role of upstream brain regions such as the premotor and parietal cortices in the pathophysiology of dystonia. In a very recent study by Merchant et al. (2020), the authors demonstrated significant interactions between the principal nodes of fine motor control namely, the ventral premotor cortex, the anterior and dorsal inferior parietal lobules, and the motor cortex by integrating TMS, structural and functional MRI methods. They found that the parieto-motor interactions as assessed by TMS were abnormal in WC patients. Although there was no significant change in the structural connectivity within the parietal-premotor-motor network in these patients, the dorsal inferior parietal lobule-premotor connectivity in the resting state was abnormally high in them. By suppressing the activity of the dorsal inferior parietal lobule using continuous theta burst stimulation, the parieto-motor interactions were restored to levels similar to healthy controls. The findings of this study indicate that the dorsal inferior parietal lobule, a region that is crucial for multimodal sensory association, could be interfering with the fine motor control network in WC patients and the same can possibly be restored by appropriate non-invasive brain stimulation methods. de Vries et al. (2012) studied the impact of low-frequency rTMS over the left superior parietal cortex on the fMRI activation patterns during executed and imagined wrist movement in cervical dystonia patients. Cervical dystonia patients showed similar but weaker activation patterns especially in the angular gyrus, suggesting poor compensatory ability of the superior parietal cortex in these patients. To add more evidence to the hypothesis of impaired compensatory mechanisms, Odorfer et al. (2019) used continuous theta burst stimulation over bilateral cerebellum to interfere with finger-tapping ability in cervical dystonia patients. They reported that finger movements, although clinically unaffected in these patients, were associated with increased activation of the lateral cerebellum on fMRI, which is likely due to compensatory disinhibitory effect on the Purkinje cells, resulting in inhibition of cerebello-thalamo-cortical circuits in cervical dystonia. Another study in adductor spasmodic dysphonia patients also suggests a possible imbalance of inhibitory processes during phonation and its correlation with hemodynamic activation of the left laryngeal motor cortex on fMRI (Chen et al., 2020). Lumsden et al. (2015) studied children with acquired dystonia in whom they performed diffusion tensor imaging (DTI) and also measured central motor conduction time (CMCT) using TMS. They found that over half of the patients had normal CMCT in spite of white matter damage. Moreover, CMCT in these patients did not correlate with DTI parameters, and, also, changes in CMCT were not reflected as changes in DTI measures. This implies that the pathology involved disruptions in the sensory connections rather than in the corticospinal tract (Mcclelland et al., 2011). Studies using multimodal approaches other than TMS-MRI, such as TMS-M/EEG could be more informative but are very few.

Thirugnanasambandam et al. (2021) aimed at exploring the neurophysiological mechanisms that underlie sensory trick in cervical dystonia patients using concurrent TMS and EEG. The study results reveal a long-latency component of TMS-evoked potential from primary motor cortex stimulation that correlated with disease duration and was exclusively present only in cervical dystonia patients who exhibited an effective sensory trick. This component, which corresponds to cortical excitation levels, is reduced during a sensory trick in patients thereby, implying that the sensory trick is likely to occur from the reduction of abnormal cortical facilitation observed in cervical dystonia patients.

## Discussion

**FIGURE 2:**
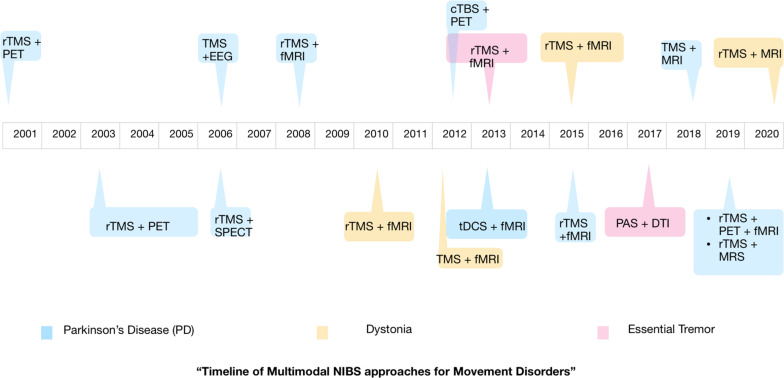
Timeline showing the publication of multimodal non-invasive brain stimulation (NIBS) studies investigating movement disorders. Parkinson’s disease is the most studied movement disorder using multimodal NIBS. The majority of studies used a combination of repetitive TMS and MRI.

From this mini-review, the dearth of multimodal NIBS studies conducted in movement disorders patients, especially ET and dystonia, is clearly evident. Figure 2 shows the timeline of the various multimodal NIBS studies investigating movement disorders that have been published up to now. Although NIBS and neuroimaging studies independently have been useful in our understanding of disease pathophysiology, well-designed multimodal NIBS studies can yield a wealth of new information that may not be obtained from these methods used in isolation. There is growing evidence in the literature to support the notion that movement disorders are associated with aberrant cortical and subcortical functional connectivity (Poston and Eidelberg, 2012). Integrating non-invasive brain stimulation and functional neuroimaging methods could be an ideal approach to investigating these connectivity changes and possibly restoring normal connectivity in patients with movement disorders (Brittain and Cagnan, 2018). Particularly, tACS seems to be a promising modality to modulate functional connectivity and therefore could have therapeutic implications for movement disorders (Brittain et al., 2013; Schreglmann et al., 2021). The field of NIBS is progressing rapidly, and more innovative techniques and novel implications of existing techniques are being introduced (di Biase et al., 2019). Multimodal NIBS approaches can enrich our knowledge on the pathophysiology of movement disorders by discerning the causal role of specific brain regions in the disease pathophysiology and outlining the changes in functional brain connectivity that contribute to the disease process. This is likely to help us in developing new diagnostic tools and treatment strategies for movement disorders.

However, the multimodal NIBS approach is not without challenges and pitfalls, which may account for the low number of studies performed. One of the main challenges of combining NIBS with functional neuroimaging methods is the issue of artifacts. Although there are novel developments in the data acquisition and analysis methods, they are not without shortcomings and therefore require judicious implementation by investigators. The combination of methods should also be carefully chosen so as to best answer the primary research question. Moreover, the experiments should be designed carefully with appropriate controls to rule out any confounders from irrelevant cortical responses (Conde et al., 2019; Rocchi et al., 2021). Although the multimodal NIBS approach is likely to yield a wealth of information, unless these data are properly channeled and results carefully interpreted, there are high chances of misinterpretation or overinterpretation of the results. Recent studies have highlighted the potential of machine learning algorithms to extract hidden information from NIBS data that may also prove beneficial (Schreglmann et al., 2021). These challenges could best be overcome by having a multidisciplinary team with both clinical and technical expertise.

In summary, it is obvious that the potential of multimodal NIBS has not been adequately leveraged for the study of movement disorders due to several challenges associated with this approach. The movement disorders community should capitalize on the immense potential that novel multimodal NIBS approaches offer and exploit them to their fullest. This could be made possible by fostering multidisciplinary collaborations.

## Author Contributions

NT: conception, first draft, and review. SS: execution, first draft, and review. Both authors contributed to the article and approved the submitted version.

## Conflict of Interest

The authors declare that the research was conducted in the absence of any commercial or financial relationships that could be construed as a potential conflict of interest.

## Footnotes

1 http://pubmed.ncbi.nlm.nih.gov
